# Chemistry and pharmacology of the herb pair *Flos Lonicerae japonicae-Forsythiae fructus*

**DOI:** 10.1186/s13020-015-0044-y

**Published:** 2015-07-02

**Authors:** Yi-ping Guo, Li-gen Lin, Yi-tao Wang

**Affiliations:** State Key Laboratory of Quality Research in Chinese Medicine, Institute of Chinese Medical Sciences, University of Macau, Avenida da Universidade, Macau, SAR China

## Abstract

The Chinese medicine herb pair Flos Lonicerae japonicae (FLJ) and Forsythiae fructus (FF), is a representative *heat-clearing* (*qing re*) and *detoxifying* (*jie du*) combination that exhibits many pharmacological activities, including antibacterial, antiviral, antitumor, anti-inflammatory, and antioxidant effects. Extensive phytochemical studies have identified a series of bioactive compounds, such as chlorogenic acid from FLJ and forsythoside A from FF. This article provides a comprehensive review on the chemical and pharmacological principles of the traditional functions of FLJ-FF, and sheds light on further developments of this herb pair.

## Introduction

Although Chinese medicine (CM) often uses multicomponent formulations and the actions of each component on multiple targets [1], the use of herb pairs—the unique clinical combination of two relatively fixed herbs—is the simplest and most fundamental form of multiherb therapy aimed at specific efficacy. The role of herb pairs has been explained by the *yin* and *yang* and five-phase theories [2], and by reference to the broader philosophical and cultural frameworks that emphasize balance between bodily functions and environmental conditions [3]. Herb pairs are simpler in composition than complete formulae but still therapeutically effective. There are several aims and principles of herbal compatibility, sometimes called the “seven relations of CM”: singular application, mutual promotion, mutual assistance, mutual restraint, mutual detoxification, mutual inhibition, and mutual intoxication [4]. The principle of mutual promotion explains why herb pairs have significantly better pharmacological efficacy than individual herbs, a principle that is applied in many famous herb formulae, such as *Yinqiao* San [1]. Recent pharmacological investigation has clarified this mutual effect [5].

The herb pair of Flos Lonicerae japonicae (FLJ) and Forsythiae fructus (FF) has been widely used to cure febrile illness (*e.g.*, cold and flu) at the primary stage [6]. FLJ is the flower bud of *Lonicera japonica* Thunb and FF is the dried fruit of *Forsythia suspensa. Yinqiao* San, which contains FLJ and FF with a crude weight ratio of 1:1, is used for detoxification and relieving internal heat and fever [7]. Nowadays, the various available dosage forms of FLJ-FF herb pair, such as capsules, powder, oral decoctions, and granules, are mainly indicated for cold, fever, and even upper respiratory tract infection [8]. The most familiar ones are Vc *Yinqiao* tablet and *Shuanghuanglian* oral decoction [9]. However, the mechanisms of the formulae have not been completely elucidated, and research on this combination has rarely been summarized. This article aims to provide a comprehensive and up-to-date review of phytochemical and pharmacological studies of FLJ and FF.

## Review

### Ethnopharmacological use of the *Yinqiao* herb pair

The FLJ-FF herb pair is described as *light* (*qing*) and *floating* (*fu*), and able to clear *heat*, combat swelling, and cure boils [10, 11]. It has been widely used as an antipyretic, antidotal, and anti-inflammatory agent for the treatment of infections such as acute nephritis and erysipelas.

### Chemical constituents of FLJ and FF

More than 140 compounds have been isolated from FLJ, including flavonoids, iridoids, organic acids, and saponins. Compounds identified from FLJ are listed in Table 1, including chlorogenic acid, luteolin, loganin, and loniceroside A. One kind of important chemical component of FLJ is its volatile oil; hexadecanoic acid, octadecadienoic acid, ethyl palmitate, and dihydrocarveol are the main fatty acids identified through gas chromatography–mass spectrometry [12].

**Table 1:** Chemical constituents of *Lonicera japonica* Thunb

No.	Compounds	Resource	Ref.
Organic acids			
1	chlorogenic acid	Whole plant	[39]
2	isochlorogenic acid	Whole plant	[39]
3	caffeic acid	Flower	[57]
4	hexadecanoic acid	Whole plant	[60]
5	myristic acid	Whole plant	[60]
6	3,5-*O*-dicaffeoylquinic acid	Whole plant	[61]
7	4,5-*O*-dicaffeoylquinic acid	Whole plant	[61]
8	3,4-*O*-dicaffeoylquinic acid	Whole plant	[61]
9	1,3-*O*-dicaffeoylquinic acid	Whole plant	[61]
10	3-ferulicoylquinic	Whole plant	[61]
11	4-ferulicoylquinic	Whole plant	[61]
12	5-*O*-caffeoylquinic acid	Whole plant	[62]
13	4-*O*-caffeoylquinic acid	Whole plant	[62]
14	caffeoyl-CH_2_-*O*-quinic acid	Whole plant	[62]
15	1,5-*O*-dicaffeoylquinic acid	Whole plant	[62]
16	1,4-*O*-dicaffeoylquinic acid	Whole plant	[62]
17	methylated dicaffeoylquinic acid	Whole plant	[62]
18	oleanolic acid 28-α-*O*-L-rhamnopyranosyl-(1 → 2)-[β-δ-dxylopyranosyl(1 → 6)]-β-δ-glu-copyranosyol ester	Flowers	[57]
19	3,5-*O*-dicaffeoylquinic acid	Flower buds	[63]
20	methyl chlorogenate	Flower buds	[63]
21	3-*O*-caffeoylquinic acid butyl ester	Flower buds	[64]
22	3-*O*-caffeoylquinic acid	Flower buds	[65]
23	3-caffeoylquinic acid methyl ester	Flower buds	[65]
24	3,5-dicaffeoylquinic acid butyl ester	Flower buds	[65]
25	vanillic acid 4-*O*-β-δ-(6-*O*-benzoylglucopyranoside)	Flower buds	[63]
26	protocatechuic acid	Flowers	[39]
27	chlorogenic acid butyl ester	Flower buds	[35]
28	chlorogenin tetraacetate	Flower buds	[66]
29	5-feruloylquinic acid	Aerial Parts	[67]
30	methyl 3,5-di-*O*-caffeoylquinic acid	Whole plant	[36]
31	methyl 3,4-di-*O*-caffeoylquinic acid	Whole plant	[36]
32	caffeic acid methyl ester	Whole plant	[37]
Flavonoids			
33	chrysoeriol	Flowers	[57]
34	chrysoeirol-7-O-neohesperidoside	Aerial parts	[57]
35	luteolin	Flowers	[57]
36	chrysoeriol 7-*O*-β-δ-glucopyranoside	Flowers	[57]
37	isorhamnetin 3-*O*-β-δ-glucopyranoside	Flowers	[57]
38	isorhamnetin 3-*O*-β-δ-rutinoside	Flowers buds	[35]
39	kaempferol 3-*O*-β-δ-glucopyranoside	Flowers	[57]
40	kaempferol 3-*O*-β-δ-rutinoside	Flowers buds	[35]
41	quercetin 3-*O*-β-δ-glucopyranoside	Flowers	[68]
42	luteolin 7-*O*-α-δ-glucoside	Flowers	[68]
43	luteolin-7-*O*-β-δ-galactoside	Flowers	[68]
44	hyperoside	Aerial parts	[69]
45	lonicerin	Whole plant	[70]
46	hydnocarpin	Aerial parts	[71]
47	quercetin	Aerial parts	[71]
48	astragalin	Aerial parts	[71]
49	isoquercitrin	Aerial parts	[71]
50	rhoifolin	Aerial parts	[71]
51	flavoyadorinin-B	Aerial parts	[63]
52	rutin	Flowers buds	[64]
53	tricin-7-*O*-β-d-glucoside	Flowers buds	[64]
54	chrysin	Leaves	[72]
55	eriodictyol	Aerial parts	[69]
56	apigenin	Aerial parts	[69]
57	corymbosin	Aerial parts	[60]
58	5-hydroxy-3, 4,7-trimethoxylflavone	Aerial parts	[60]
59	ochnaflavone	Whole plant	[71]
60	ochnaflavone 4′-*O*-methyl ether	Aerial parts	[71]
Iridoids			
61	loganin	Whole plant	[70]
62	sweroside	Flower buds	[73]
63	7-*O*-ethyl sweroside	Flower buds	[74]
64	7-epivogeloside	Flower buds	[74]
65	secoxyloganin	Flower buds	[74]
66	secoxyloganin 7-butyl ester	Flower buds	[74]
67	7-dimethyl-secologanoside	Flower buds	[74]
68	centauroside	Flower buds	[74]
69	secologanic acid	Flower buds	[62]
70	secologanin	Flower buds	[73]
71	secologanin dimethyl acetal	Flower buds	[73]
72	kingiside	Flower buds	[75]
73	vogeloside	Flower buds	[76]
74	epi-vogeloside	Flower buds	[76]
75	dehydrormorroniside	Flower buds	[77]
76	ketologanin	Flower buds	[78]
77	7α-morroniside	Flower buds	[78]
78	7β-morroniside	Flower buds	[78]
79	secologanoside	Flower buds	[78]
80	lonijaposide A	Flower buds	[78]
81	lonijaposide B	Flower buds	[78]
82	lonijaposide C	Flower buds	[78]
83	lonijaposide D	Flower buds	[78]
84	lonijaposide E	Flower buds	[78]
85	lonijaposide F	Flower buds	[78]
86	lonijaposide G	Flower buds	[78]
87	lonijaposide H	Flower buds	[78]
88	lonijaposide I	Flower buds	[78]
89	lonijaposide J	Flower buds	[78]
90	lonijaposide K	Flower buds	[78]
91	lonijaposide L	Flower buds	[78]
92	l-phenylalaninosecologanin	Stems,leaves	[79]
93	7-*O*-(4-β-d-glucopyranosyloxy-3-methoxy-benzoyl) secologanolic acid	Stems,leaves	[79]
94	6′-*O*-(7α-hydroxyswerosyloxy) loganin	Stems,leaves	[79]
95	(*Z*)-aldosecologanin	Stems,leaves	[79]
96	(*E*)-aldosecologanin	Stems,leaves	[79]
97	loniceracetalide A	Flower buds	[76]
98	loniceracetalide B	Flower buds	[76]
Saponins			
99	3-*O*-α-L-arabinopyranosyl-28-*O*-[β-d-glucopyranosyl(1 → 6)-β-dglucopyranosyl] oleanolic acid	Aerial parts	[80]
100	3-*O*-[α-L-rahmnopyranosyl(1 → 2)-α-l-arabinopyranosyl]-28-*O*-β-dglucopyran-Osyl hederagenin	Aerial parts	[80]
101	3-*O*-[α-l-rahmnopyranosyl(1 → 2)-α-l-arabinopyranosyl]-28-*O*-[β-dglucopyranosyl(1 → 6)-β-d-glucopyranosyl] oleanolic acid	Aerial parts	[80]
102	3-*O*-[α-l-rahmnopyranosyl(1 → 2)-α-l-arabinopyranosyl]-2 8-*O*-[6-acetyl-d-glucopyranosyl (1 → 6)-α-d-glucopyranosyl] hederagenin	Aerial parts	[80]
103	3-*O*-α-l-rhamnopyranosyl-(1 → 2)-α-l-arabinopyranosy hederagenin28-*O*-β-d-xylpyranosyl (1 → 6)-β-d-glucopyranosyl ester	Flower buds	[66]
104	3-*O*-α-l-arabinopyranosy hederagenin 28-*O*-α-d-rhamnopyranosyl(1 → 2) [δ-d-xyl pyranosyl(1 → 6)-β-d-glucopyranosyl ester	Flower buds	[66]
105	3-*O*-α-l-rhamnopyranosyl-(1 → 2)-α-l-arabinopyranosy hederagenin28-*O*-β-d- rhamnopyranosyl(1 → 2)[β-d-xylpyranosyl(1 → 6)-β-d-glucopyranosyl ester	Flower buds	[66]
106	3-*O*-β-d-glucopyranosyl-(1 → 4)-β-l-glucopyranosyl(1 → 3)-α-lrhamnopyranosyl (1 → 2)-α -l-arabinopyranosyhederagenin28-*O*-β-d-glucopyranosyl-(1 → 6)-β-d-glucopyranosyl ester	Flower buds	[81]
107	Hederagenin-3-*O*-α-l-rhamnopyranosyl(1 → 2)-α-larabinopyranoside	Flower buds	[81]
108	3-*O*-α-l-rhamnopyranosyl-(1 → 2)-α-l-arabinopyranosy hederagenin28-*O*-β-d-glucopyranosyl (1 → 6)-β-d-glucopyranosyl ester	Flower buds	[81]
109	3-*O*-β-d-glucopyranosyl-(1 → 3)-α-l-rhamnopyranosyl(1 → 2)-α-larabino pyranosyl hederagenin 28-O-β-d-glucopy ranosyl-(1 → 6)-β-d-glucopyranosyl ester	Flower buds	[81]
110	loniceroside A	Whole plant	[75]
111	loniceroside B	Whole plant	[75]
112	loniceroside C	Aerial parts	[82]
113	loniceroside D	Flower buds	[83]
114	loniceroside E	Flower buds	[83]
115	macranthoidin A	Flower buds	[64]
116	macranthoidin B	Flower buds	[64]
117	dipsacoside B	Flower buds	[64]
118	hederagenin-28-*O*-[β-d-glucopyranosyl-(1 → 6)-β-d-glucopyranosyl] ester	Flower buds	[64]
119	macranthoside B	Flower buds	[64]
120	macranthoside A	Flower buds	[64]
121	3-*O*-[α-l-rhamnopyranosyl-(1 → 2)-α-l-arabinopyranosyl] hederagenin	Flower buds	[64]
122	saponin 1	Flower buds	[62]
123	saponin 4	Flower buds	[62]
124	hederagenin 3-*O*-α-l-arabinopyranoside	Flowers	[57]
125	hederagenin	Whole plant	[70]
126	oleanolic acid	Flower buds	[35]
Others			
127	lonijaposide A1	Flowers	[72]
128	lonijaposide A2	Flowers	[72]
129	lonijaposide A3	Flowers	[72]
130	lonijaposide A4	Flowers	[72]
131	lonijaposide B1	Flowers	[72]
132	lonijaposide B2	Flowers	[72]
133	5-hydroxymethyl-2-furfural	Flowers	[57]
134	1-*O*-methyl-myo-inositol	Flower buds	[35]
135	nonacontane	Flower buds	[35]
136	β-sitosterol	Flower buds	[35]
137	sucrose	Flower buds	[35]
138	glucose	Flower buds	[35]
139	shuangkangsu	Flower	[59]
140	(+)-N-(3-methybutyryl-β-d-glucopyranoyl)-nicotinate	Flower buds	[78]
141	(+)-N-(3-methybut-2-enoyl-β-d-glucopyranoyl)-nicotinate	Flower buds	[78]
142	5′-*O*-methyladenosine	Flower buds	[78]
143	guanosine	Flower buds	[78]
144	adenosine	Flower buds	[78]
145	syringin	Flower buds	[78]

More than 100 compounds have been identified from FF, including alkaloids, flavonoids, phenylethanoid glycosides, triterpenoids, and lignans (Table 2). The major constituents of FF include quercetin, rutaecarpine, forsythiaside A, betulinic acid, and forsythialan A. According to the China Pharmacopoeia (2005 edition), forsythin is a chemical marker for quality control of FF. Recent pharmacological research indicates that forsythiaside, forsythin, and rutin are responsible for the biological activities of FF [13]. Thus, the use of forsythin as a single marker for FF quality control is likely to lead to biased assessment. Quantification of both forsythiaside and forsythin in FF is important for the evaluation of this herb’s quality. In the 2010 edition of the China Pharmacopoeia, both forsythin and forsythiaside were specified as FF quality control markers.

**Table 2:** Chemical constituents of *Forsythiae fructus*

No.	Compounds	Ref.
Phenylethanoid Glycosides		
146	forsythoside A	[84]
147	forsythoside B	[85]
148	forsythoside C	[85]
149	forsythoside D	[85]
150	forsythoside E	[86]
151	forsythoside F	[86]
152	isoforsythoside	[87]
153	forsythoside H	[86]
154	forsythoside I	[86]
155	forsythoside J	[86]
156	*R*-forsythoside J	[88]
157	*S*-forsythoside J	[88]
158	*R*-suspensaside	[88]
159	*S*-suspensaside	[88]
160	*S*-suspensaside methyl ether	[88]
161	calceolarioside A	[89]
162	calceolarioside B	[86]
163	plantainoside A	[89]
164	suspensaside A	[90]
165	lianqiaoxinoside B	[54]
166	salidroside	[91]
167	3,4-dihydroxyphenyl alcohol-8-o-β-D-glucopyranoside	[90]
168	forsythenside A	[35]
169	forsythenside F	[35]
170	phenethylalcohol-β-D-xylopyranosyl-(1–6)-β-D-glucopyranoside	[35]
Triterpenoids		
171	isobauerenyl acetate	[90]
172	ocotillone	[90]
173	ocotillol acetate	[90]
174	20(*S*)-dammar-24-ene-3β,20-diol-3-acetate	[92]
175	oleanolic acid	[93]
176	β-amyrinacetate	[92]
177	ursolic acid	[93]
178	2α,3α-hydroxyursolic acid	[94]
179	2α,23-hydroxyursolic acid	[93]
180	betulinic acid	[95]
181	3β-acetylbetulinic acid	[95]
182	2α-hydroxybetulinic acid	[93]
183	3β*-*acetyl-20,25-epoxydammarane-24α-ol	[96]
184	3β-acetyl-20,25-epoxydammarane-24β-ol	[96]
185	onjisaponin F	[94]
186	onjisaponin G	[94]
Lignans		
187	(−) arctigenin	[97]
188	(−) dimethylmatairesinol	[97]
189	(+) phillygenin	[93]
190	(+) phillyrin (forsythin)	[98]
191	(+) epipinoresinol	[88]
192	(+) epipinoresinol-4-*O*-β-D-glucoside	[98]
193	(+) epipinoresinol-4′-*O*-β-D-glucoside	[88]
194	(+)-1-hydroxy-6-epipinoresinol	[88]
195	(+)-1-hydroxy-6-epipinoresinol-4″-*O*-β-D-glucopyranoside	[88]
196	7′-*epi*-8-hydroxypinoresinol	[88]
197	(+) pinoresinol	[88]
198	(+) pinoresinol-β-D-glucoside	[99]
199	(+) pinoresinol monomethyl ether-β-D-glucoside	[99]
200	(+)-1-hydroxypinordsinol	[88]
201	(+)-1-hydroxypinordsinol-4′-*O*-β-D-glucoside	[88]
202	(+)-1-hydroxypinordsinol-4″-*O*-β-D-glucoside	[88]
203	8-hydroxypinoresinol	[55]
204	isolariciresinol	[55]
205	isolariciresinol-4-*O*-β-D-glucopyranoside	[100]
206	isolariciresinol-9′-*O*-β-D-glucopyranoside	[100]
207	(+)-isoolivil	[100]
208	cedrusin	[100]
209	benzenebutanoic acid	[55]
210	olivil	[97]
211	(+) lariciresinol	[55]
212	forsythialan A	[101]
213	forsythialan B	[101]
Iridoid Glycosides		
214	adoxosidic acid	[90]
C6-C2 Natural Alcohols		
215	rengyol	[102]
216	isorengyol	[102]
217	rengyoside A	[91]
218	rengyoside C	[91]
219	suspenol	[84]
220	rengyolester	[103]
221	rengynic acid	[98]
222	rengynic acid 1′-*O*-β-D-glucoside	[98]
223	rengyoxide	[104]
224	rengyoside B	[91]
225	rengyolone	[91]
226	cornoside	[104]
227	forsythenside B	[90]
Flavonoids		
228	quercetin	[105]
229	hyperin	[106]
230	rutin	[105]
231	wogonin-7-*O*-glucoside	[98]
232	hesperidin	[106]
Alkaloids		
233	rutaecarpine	[107]
234	suspensine A	[108]
235	(−)-egenine	[108]
236	(−)-7′-*O*-methylegenine	[108]
237	(−)-bicuculline	[108]
238	octahydro-1H, 5H-dipyrrolo [1,2-a:1′,2′-d] pyrazine	[109]
Others		
239	stearic acid	[16]
240	palmitic acid	[16]
241	vanillic acid	[90]
242	caffeic acid	[90]
243	4-methoxycaffeic acid	[100]
244	chlorogenic acid	[106]
245	4-caffeoylrutinose	[98]
246	suspenolic acid	[98]
247	3-ethyl-7-hydroxyphthalide	[67]
248	forsythenin	[90]
249	tannic acid	[110]

### Pharmacological effects

Forsythoside A has strong antioxidant, antibacterial, and antiviral activities [14]. Forsythiaside exhibits strong antibacterial, antiviral, antioxidant, anti-inflammatory, and cyclic adenosine monophosphate phosphodiesterase inhibitory effects [15]; forsythin and rutin show a strong antioxidant effect [16]. In addition, chlorogenic acid possesses antibacterial and antiviral activities [17]. The bioactive properties of FLJ and FF are summarized below in terms of traditional functions and modern pharmacological findings.

#### Effects of the FLJ-FF herb pair

Studies of the synergistic action of the FLJ-FF herb pair are rare. There is little evidence for its synergistic properties, although Li *et al.* [18] reported a synergistic anti-inflammatory effect. They established a rat model of chronic obstructive pulmonary disease and treated the animals with FLJ-FF herb pair extract (FLJ:FF, 2:3) and a single herb extract. FLJ-FF herb pair treatment improved chronic obstructive pulmonary disease pathological changes and significantly reduced interleukin-1β (IL-1β) levels in bronchoalveolar lavage fluid compared with each single herb. Duan *et al.* [19] evaluated anti-free radical activity of the FLJ-FF herb pair using a rat fever model. They divided Sprague–Dawley rats into different groups, treated some groups with 20 % dilute yeast suspension to create a fever model, and then tested the effectiveness of different drug combinations. The FLJ-FF herb pair showed potent free radical cleavage activity. Using microbial-plate methods, Wang *et al.* [20] found the FLJ-FF herb pair at the ratio of 1:6 showed the most significant inhibitory effect on *Streptococcus suis* 2. These studies indicate that FLJ and FF show stronger bioactivity in combination than alone.

#### Detoxifying: antibacterial action

FF is a broad-spectrum antimicrobial agent used mainly for upper respiratory tract infection and acute nephritis [10, 11]. Previous studies have shown that the antibacterial ingredients of *Forsythia* species are concentrated in lignans, phenylethanoid glycosides, and volatile oil. Endo *et al.* [21, 22] investigated the antibacterial principle of *F. suspensa* leaves, and found that forsythosides A, B, C, and D showed antibacterial activity against *Staphylococcus aureus* at <2 μM. In 2005, the effect of *F. suspensa* volatile oil against *Saccharomyces cerevisiae*, *Penicillium chrysogenum*, and *Aspergillus niger* was estimated by paper dispersion method*.* The minimal bactericidal concentrations (MBC) were 3.91 × 10^−4^ mL/100 mL, 7.81 × 10^−4^ mL/100 mL, and 3.13 × 10^−3^ mL/100 mL, respectively [23]. Chen’s group [11] investigated the therapeutic effect of FF ethanol extract on *Salmonella typhimurium* infection in mice. The survival rate of mice treated by FF ethanol extract was higher than in the control group; the numbers of viable bacteria in spleen and the spleen weight index were much lower in the treatment group. Furthermore, the levels of immunoglobulin G and interferon γ increased in the treatment group, compared with the control group. The survival rate of the infected mice was as high as 70 % when the therapeutic dose of FF ethanol extract was 30 g/kg/day, which demonstrates a significant relationship between input (dose) and output (effect) (P < 0.05). All of the above studies support the traditional use of FF as an antibacterial agent.

The antibacterial activity of FLJ has been comprehensively studied. In 2009, Rahman *et al.* [12] evaluated the antibacterial potential of FLJ volatile oil, which showed a remarkable antibacterial effect against *Listeria monocytogenes* (ATCC 19116), *Bacillus subtilis* (ATCC 6633), *Bacillus cereus* SCK 111, *Staphylococcus aureus* (ATCC 6538 and KCTC 1916), *Salmonella enteritidis* (KCTC 12021), *Salmonella typhimurium* (KCTC 2515), *Enterobacter aerogenes* (KCTC 2190), and *Escherichia coli* (ATCC 8739). The inhibition zone diameters were 20.3, 17.8, 15.2, 16.3, 14.1, 15.3, 14.0, 12.4 and 12.1 mm, respectively. The MIC values were 62.5, 62.5, 250, 125, 250, 125, 250, 500 and 500 μg/mL, respectively. These findings suggest that FLJ volatile oil is a potential source of preservatives for the food or pharmaceutical industries. The antibacterial activities of FLJ against *Bacillus cereus* and *Staphylococcus aureus* were tested using the agar-well diffusion method *in vitro*. The inhibition zone diameters were 6.3 and 7.2 mm, respectively; this activity might be closely associated with the existence of phenolic constituents [24]. Moreover, FLJ exhibited marked antibacterial activity against 14 strains, including *Staphylococcus aureus, Streptococcus haemolyticus, Escherichia coli, Bacillus dysenteriae, Bacillus comma, Bacillus typhosus, Bacillus paratyphosus, Pseudomonas aeruginosa, Klebsiella pneumoniae, Bacillus tuberculosis, Streptococcus mutans, Bacillus adhaerens, Bacteroides melaninogenicus, and Haemophilus actinomycetemcomitans*. FLJ extracts have also been found to inhibit 87.5 % strains using MIC 25 mg/mL [25].

One study investigated the antimicrobial activity of FLJ water and alcohol extract. The MIC and MBC values for the water extract on *Staphylococcus aureus* were 19.25 and 38.50 %, respectively; the MIC and MBC values for the alcohol extract on *Salmonella enteritidis* were 9.80 and 19.60 %, respectively, and for *Staphylococcus aureus* were 19.60 and 39.20 %, respectively [26]. FLJ flavonoids also showed a strong antibacterial action, especially for methicillin-resistant *Staphylococcus aureus* (MIC ≤5 mg/mL) [27]. These reports suggest that FLJ is a potent agent for treating various bacteria.

#### Detoxifying: antiviral activity

Several studies have demonstrated the antiviral activity of FF. FF aqueous extract showed antiviral activity against respiratory syncytial virus (RSV) with IC_50_ 50 μg/mL and CC_50_ 1000 μg/mL [28]. Wen *et al.* found that 80 % ethanol extract of FF had a significant protective effect on Madin–Darby canine kidney cells infected by the H_1_N_1_ virus in a dose-dependent manner [29]. Li *et al.* [30] assessed the effects of forsythoside A on cell infection by avian infectious bronchitis virus; the data indicated that this compound prevented virus infection *in vitro*, but the mechanisms remain unclear.

Many studies have disclosed antiviral activity of FLJ, including anti-RSV, anti-HIV (human immunodeficiency virus), and anti-NDV (Newcastle disease virus) effects. Two studies used a cytopathologic effect (CPE) assay to test the antiviral activities against RSV of 44 medicinal herbs used for the treatment of respiratory tract infectious diseases in China [28, 31]. FLJ showed potent antiviral activity against RSV; the IC_50_ was 50.0 μg/mL and the selectivity index was more than 20.0. FLJ extract and chlorogenic acid had significant anti-cytomegalovirus activity, and the 0 % toxic dose, minimum effective concentration, and therapeutic index (TI) of these two composites for human cytomegalovirus were 3000 μg/mL, 3000 μg/mL, 1 and 100 μg/mL, 1 μg/mL, 100, respectively [32]. In *in vitro* tests, FLJ extract showed 104 and 72 times the TI for anti-herpes simplex virus-1 F and anti-herpes simplex-1 to acyclovir. Regarding the caviid beta-herpes virus 1, FLJ showed significant inhibition of the duplication of guinea pig cytomegalovirus at the cell level; the TI and the inhibitory duplication index were 100 and 2.61 μg/mL, respectively [33]. The anti-virus (H_9_N_2_) and anti-avian influenza virus (least effective dose [LED] = 3.90 mg/mL, *in vitro*) activities of FLJ flavones have also been tested [34]. In Vero cells, three different extracts of FLJ, including volatile oil (P1), chlorogenic acids extract (P2), and flavones extract (P3), were tested for antiviral activity against the pseudorabies virus (PRV) and NDV. At doses of 232.7, 116.35, 58.18, and 29.09 μg/mL, the P1 interdiction rates for PRV and NDV were 40.13 %, 17.83 %, 13.16 %, 2.24 % and 75.40 %, 32.01 %, 12.05 %, 2.34 % on the CPE, respectively, and the LEDs of P1 for PRV and NDV were 232.7 and 232.7 μg/mL, respectively. The P2 interdiction rates (3.125, 1.563, 0.781, and 0.391 mg/mL) for PRV and NDV were 63.74 %, 46.27 %, 13.10 %, 3.51 % and 65.23 %, 36.71 %, 32.61 %, 28.96 % on the CPE, respectively. The P3 interdiction rates (1.954, 0.977, 0.489, and 0.244 mg/mL) for PRV and NDV were 94.00 %, 78.42 %, 42.30 %, 3.36 % and 78.07 %, 27.63 %, 16.37 %, 6.73 %, respectively. For P2 and P3, the LEDs against PRV were 0.997 and 3.097 mg/mL, respectively and against NVD, they were 0.781 and 1.563 mg/mL, respectively. These studies suggest that FLJ extracts decrease CPE lesions and neutralize viruses in a dose-dependent manner, inhibiting viruses directly and promoting cell antivirus responses [35].

Several FLJ tannins have also been investigated; 3,5-di-*O*-caffeoylquinic acid and methyl 3,5-di-*O*-caffeoylquinic acids had a strong inhibitory effect on HIV-1 reverse transcriptase (RT) and human DNA polymerase-α (HDNAP-α) [36]. The IC_50_ ratio of these two compounds for HIV-1 RT and HDNAP-α was 2.0 and 2.2, respectively. 3,4-di-*O*-caffeoylquinic acid and methyl 3,4-di-*O*-caffeoylquinic acid exhibited higher inhibitory effects on HDNAP-α than on HIV-1 RT [36]. Thirteen other caffeoylquinic acids isolated from FLJ, including caffeic acid and caffeic acid methyl ester, were also found to show antiviral activities against respiratory viruses [37]. FLJ extract showed an obvious therapeutic action on mice infected with influenza A virus pneumonia [31]. The lung indexes of the FLJ group and the ribavirin group were significantly lower than in the model group, but there was no significance difference between the two treatment groups. FLJ extract reduced histopathological changes, viral duplication, and the contents of influenza virus nucleic acid compared with the model group. The tumor necrosis factor-α (TNF-α) and IL-1β expressions of the FLJ and the ribavirin groups were significantly lower than those of the model group. The FLJ chemical principles for antiviral activity were identified as chlorogenic acids, flavones, tannins, and volatile oil [31].

#### Detoxifying: antitumor activity

The apoptosis mechanisms induced by photodynamic therapy (PDT) in lung CH27 carcinoma cells, cultured with FLJ alcohol extract as a photosensitizer, have been explored. This extract exhibited significant photocytotoxicity in CH27 cells at a concentration range of 50–150 μg/mL, with light doses of 0.4–1.2 J/cm^2^. Apoptosis induced by PDT combined with FLJ extract was accompanied by DNA condensation, externalization of phosphatidylserine, and formation of apoptotic bodies [38]. The p38-associated pathway might be involved in apoptosis induced by PDT with FLJ in CH27 cells. In another study, FLJ extract induced CH27 cell apoptosis *via* protein expression change and distribution of heat shock protein 27. Treatment with FLJ aqueous extract (100 μg/mL) was associated with increased stimulatory phosphorylation of c-Jun amino-terminal kinase and p38 in HepG2 cells, similar to the mitogen-activated protein kinase activation profile of protocatechuic acid [39]. This aqueous extract also decreased the viability of HepG2 cells to 50 % and triggered HepG2 cell death in a c-Jun amino-terminal kinase-dependent manner.

#### Heating clearing: anti-inflammatory activity

Inflammation prevents infection through production of pro-inflammatory cytokines and generation of inflammatory mediators in response to microbial products [40]. Dysregulation of inflammation has an adverse effect on the body. Although modern anti-inflammatory drugs can bring relief, new kinds of microorganisms and the emergence of drug-resistant strains have resulted in significant morbidity and mortality. In the past few decades, more attention has been focused on the anti-inflammatory effect of CM herbs, especially *heat-clearing* herbs [41].

Many studies have demonstrated the anti-inflammatory action of FF. FF was found to exhibit platelet-activating factor antagonistic activity and inducible nitric oxide synthase inhibitory activity [42]. An FF methanol extract and its hexane fraction showed anti-inflammatory and analgesic activity against carrageenan-induced edema, cotton pellet-induced granuloma, and acetic acid-induced vascular permeability [43]. FF extract inhibited 5-lipoxygenase and elastase with the same IC_50_ values of 80 μg/mL [44]. FF ethanol extract also inhibited the secretion of the cytokine RANTES from virus-infected human bronchial epithelial cells [45]. These findings suggest that FF possesses anti-inflammatory activity through multiple target signaling pathways and multiple mechanisms of action.

Both *in vivo* and *in vitro* studies have shown that FLJ extract can inhibit various inflammatory reactions and suppress various inflammatory factors. Xu *et al.* [46] evaluated the anti-inflammatory property of FLJ aqueous extract in A549 cells; the extract directly inhibited both COX-1 and COX-2 activity, and IL-1-induced expression of COX-2 protein and mRNA. Kang *et al.* [47] examined the effect of FLJ water fraction on trypsin-induced mast cell activation. After stimulation with trypsin (100 μM), FLJ water fraction inhibited TNF-α secretion, tryptase mRNA expression, and trypsin-induced extracellular signal-regulated kinase phosphorylation in a dose-dependent manner; however, it did not affect trypsin activity even at 1000 μg/mL. These studies indicate that FLJ might inhibit trypsin-induced mast cell activation through the inhibition of extracellular signal-regulated kinase phosphorylation rather than by inhibition of trypsin activity. One study evaluated the anti-inflammatory activity of *n*-butanol (4.2 % based on the dry weight [DW]) FJL fraction [48]. At a 400 mg/kg oral dose, it showed significant anti-inflammatory activities against arachidonic acid ear edema, croton-oil ear edema, carrageenan paw edema, and rat cotton pellet granulomatous and adjuvant-induced arthritis inflammation models in mice and rats; the inhibition rates were 27 %, 23 %, 26 %, 18 %, and 42 %, respectively and the inhibition rates for the positive drug aspirin (100 mg/kg) were 27 %, 13 %, 13 %, 0 %, and 58 %, respectively.

FLJ water extract showed an anti-inflammatory effect on proteinase activated receptor 2 (PAR2)-mediated mouse paw edema; at doses of 50, 100, and 200 mg/kg, it significantly inhibited paw thickness change and vascular permeability induced by PAR2 (inhibition rates: 41.8 %, 69.1 %, 70.9 %, and 40.2 %, 69.7 %, 68.8 %, respectively). FLJ water extracts (100 mg/kg) also significantly inhibited PAR2 agonist-induced myeloperoxidase (MPO) activity and TNF-α expression in paw tissue [49]. Tae *et al.* [50] used the supercritical CO_2_ extraction process to obtain 1.08 % volatile oil from FLJ; pharmacological studies suggested a potent anti-inflammatory effect of the volatile oil on the ear-swelling model in mice. These reports indicate that FLJ is a safe, mild anti-inflammatory agent for treating various inflammatory disorders.

#### Heat clearing: antioxidant activity

Excessive reactive oxygen species result in significant damage to biological structures necessary to cellular integrity and survival. CM *heat-clearing* herbs are an important source of antioxidant agents. A study using a 1-diphenyl-2-picrylhydrazyl (DPPH) free radical scavenging experiment found that the CH_2_Cl_2_ fraction of *F. suspensa* exerted the strongest scavenging activity and suggested that forsythialan A and phillygenin F are the major antioxidant constituents [51, 52]. Zhang *et al.* [53] studied the role of forsythoside A in the elimination of reactive oxygen species and discussed the relationship between structure and activity using quantum chemical calculation. The results showed that the A and B rings in forsythoside A were active parts of its antioxidant activity, and the structure of phenolic hydroxyl groups in *opposition* caused higher antioxidant activity. Moreover, lianqiaoxinoside B and forsythoside H showed nearly the same antioxidant activities. These phenylethanoid glycosides have two ortho-substituting hydroxyl groups in both the caffeoyl and phenylethanoid moieties, which could be an important factor in their high antioxidant activity [54]. Lignans obtained from FF could protect human high-density lipoprotein against lipid peroxidation. In one study, they inhibited the generation of thiobarbituric acid-reactive substances in a dose-dependent manner with IC_50_ values from 8.5 to 18.7 μM. Among these lignans, some exerted an inhibitory effect against the Cu^2+^-induced lipid peroxidation of high-density lipoprotein, as shown by an extended lag time prolongation at a 3.0 μM concentration [55]. The protective activity of *F. suspensa* against peroxynitrite (ONOO)-induced cellular damage was investigated, and its active components, phillygenin and 8-hydroxypinoresinol, were identified. These two compounds significantly reduced cell injury by 3-morpholinosydnonimine, an ONOO generator. The hydroxyl substituents of these lignans on the phenyl moieties may contribute to the antioxidant activity [56].

The antioxidant action of FLJ has been widely investigated. The FLJ ethyl acetate fraction exhibited marked scavenging/inhibitory activities with IC_50_ values of 4.37, 27.58 ± 0.71, 0.47 ± 0.05, and 12.13 ± 0.79 μg/mL in the DPPH radical, total reactive oxygen species, hydroxyl radical (−OH), and peroxynitrite (ONOO^−^) assays, respectively [57]. The main compounds of the ethyl acetate fraction—luteolin, caffeic acid, protocatechuic acid, and luteolin 7-*O*-d-glucopyranoside—also evidenced marked scavenging activities, with IC_50_ values of 2.08–11.76 μM for DPPH and 1.47–6.98 μM for ONOO^−^ [57]. The Trolox equivalent antioxidant capacity values and total phenolic content for methanolic extracts of FLJ have been demonstrated as 589.1 μmol Trolox equivalent/100 g DW and 3.63 gallic acid equivalent/100 g DW [58]. These studies suggest that FLJ is a potential natural antioxidant and beneficial chemopreventive agent. The antioxidant activity of polysaccharides with different molecule weights separated from FLJ by ultrafiltration was also studied. The reducing power of the polysaccharides had a direct correlation with antioxidant activity and concentration of certain plant extracts, and the ultrafiltration fraction had a significant inhibitory effect on superoxide radicals generated in a phenazine methosulphate/hydrogenated nicotinamide adenine dinucleotide/nitroblue tetrazolium system. Administered to rats, crude polysaccharide extracts (50–400 mg/kg) were found to reduce lipid peroxidation malondialdehyde content, improve glutathione peroxidase and catalase activity, and significantly enhance superoxide dismutase activity in serum and tissue [59].

### Limitations of this review

Few studies demonstrated a synergistic or additive effect for this herb pair. Comparable studies, using both single herbs (FLJ and FF) and the FLJ-FF herb pair, should be conducted to investigate possible synergistic or additive effects. Interdisciplinary research is needed to identify minor bioactive components using phytochemical studies, to generate reliable cell and animal models using pharmacological studies, and to elucidate underlying mechanisms using molecular biological studies.

All pharmacological studies reviewed here used *in vitro* or *in vivo* models; there was no clinical investigation of the effects of the FLJ-FF herb pair (or of single herbs). Thus, this review provides no clinical evidence for the bioactivities of FLJ and FF. In addition, some of the pharmacological targets of the FLJ-FF herb pair are still unknown.

## Conclusion

The main bioactive components of FLJ and FF are flavonoids, organic acids, volatile oil, phenylethanoid glycosides, lignans, and triterpenoids. These show clear pharmacological effects, including antibacterial, antiviral, anti-inflammatory, antitumor, and antioxidant actions.

## Abbreviations

CM: Chinese Medicines
FLJ: Flos Lonicerae japonicae
FF: Forsythiae fructus
MIC: Minimum inhibitory concentration
MBC: Minimum bactericidal concentration
RSV: Respiratory syncytial virus
HIV: Human Immunodeficiency virus
NDV: Newcastle disease virus
CPE: Cytopathologic effect
SI: Selectivity index
TNF-α: Tumor necrosis factor-α
IL-1β: interleukin-1β
TI: Therapeutic index
PDT: Photodynamic therapy
LED: Least effective dose
PRV: Pseudo rabies virus
PAR2: Proteinase activated receptor 2
MPO: Myeloperoxidase
DPPH: 1-diphenyl-2-picrylhydrazyl
